# Quantification of memory effects in topological two-band open quantum systems

**DOI:** 10.1016/j.heliyon.2024.e40552

**Published:** 2024-11-20

**Authors:** H. Triviño, F. Mesa, VA. Ballesteros

**Affiliations:** a Universidad de Antioquia, Facultad de Ciencias Exactas y Naturales, Grupo de Investigación en Física Teórica y Matemáticas Aplicadas, Calle 70 No. 52-21, Medellín, 050010, Antioquia, Colombia; b Fundación Universitaria Los Libertadores, Facultad de Ingeniería y Ciencias Básicas, NanoTech Group, Cra.16 No. 63a-68, Bogotá, 111221, Cundinamarca, Colombia

**Keywords:** Non-Markovianity, Two-band model, Memory effects, Nonlinear spectroscopy

## Abstract

We incorporate non-Markovian profiles and Linear Response Theory to analyze memory effects in two-band topological quantum systems. Furthermore, we have applied a measure of non-Markovianity in terms of nonlinear optical spectroscopy. On the other hand, we resort to memory kernel, solve the integro-differential equation of the open two-band topological quantum system to describe the degrees of non-Markovianity, calculate response factors based on Linear Response Theory, and analyze non-Markovian dynamics by varying the parameters of the nonlinear spectroscopy environment of the respective open quantum system.

## 1 Introduction

Markovian open quantum systems, have been intensively studied for fundamental and applied reasons. From a fundamental point of view, the study of quantum systems interacting with structured environments (reservoirs) presents considerable difficulties from a theoretical point of view [1], [2], [3], [4], [5]. However, from an applied point of view in the field of solid-state physics [6], [7], [8], biochemistry and quantum technologies [7], [8], it opens the way to new methods of decoherence control based on the manipulation and modification of the properties of the environment [8], [9], [10], such as its frequency spectrum [2], [10].

Now, given an open quantum system (system-reservoir) in a separable complex Hilbert space, there are different measures to easily verify the degree of Markovianity or non-Markovianity (memory effects) [11], [12], [13], [14]. However, many questions remain to be studied. In this case, fundamental questions such as the mathematical structure of non-Markovian quantum dynamics [15], [16], the role of complexity in the occurrence of memory effects [17], [18], [19], [20], [21], [22], or the relevance of non-Markovianity in the study of the boundary between classical and quantum aspects, as well as more applied questions such as the identification of environmental features or the correlation between the system and the environment [23], [24], [25], [26], [27], [28], [29], [30].

From another point of view, the study of non-Markovianity has also been supported by Linear Response Theory (*LRT*), which is based on first-order perturbation theory for a system in thermal equilibrium, which is one of the most useful methods to connect physical quantities with the underlying theoretical description of a system. Recently, the *LRT* was developed for open quantum systems, taking the non-Markovian effect [15]. There, a description of a system coupled to a given environment in an external field was performed, and the susceptibility in the non-Markovian regime was derived. The obtained results were applied to quantum materials theory and derived the Hall conductance for a topological insulator configured by the two-band model in the non-Markovian regime [12], [15], [29].

The two bands can describe different physics; for example, they can describe spin-orbit coupling when the system is a spin − 1/2 electron group in solids or orbital hybridization when the two bands represent orbitals and topological insulators. The derivations of the Hall conductance are completely independent of the physical explanation of the respective Hamiltonian one, so the result can be applied in many cases where the system is described by the two-band model. The system can be realized with ultracold atoms in condensed matter with different techniques, for example, superlattices and spin − 1/2 electrons with spin-coupling orbitals [15], [31], [32], [33], [34], [35].

In this case, existing experiments in the literature have demonstrated the variability of the Hall conductance configured by the two-band model from the Markovian to the non-Markovian profile. However, the presented formalism does not allow for showing all the relationships between response functions and memory effects [12], [15], [29].

Therefore, we introduce different measures of memory effects in an open quantum system configured by the two-band system (topological insulators) in terms of temperature and reservoir parameter control, mediated by Linear Response Theory, comparing the degree of non-Markovianity for the respective dynamics [1], [13], [15]. Furthermore, we turn to the solution of the integral equation of the memory kernel associated with the Lorentzian spectral density [3], [16], [19], discussing non-Markovian quantum coherence and defining a measure of the non-Markovian character in terms of a cross-correlation between the measured spectrum and a simulated Markovian spectrum [23], [36], [37].

## 2 Non-Markovianity measurements

The theory of open quantum systems applied different techniques to quantify memory effects [1], [5], [23]. In fact, numerous investigations have closed gaps between experiments and theoretical formalizations [2], [3], [13]. However, the computational cost [3], [5], the restrictions on analytical functions [2], [33], [38], and the incorporation of incoherent dynamic maps imply a loss of information in the reservoir system [33], [34], [35], [36]. In this section, we show the methods to quantify the open two-band quantum system (topological insulators) in the non-Markovian regime [1], [2], [33]. Also, we include sections on Linear Response Theory and Nonlinear Optics Spectroscopy [2], [3], [39].

### 2.1 Coherence measure

Recently, it has been shown that quantum coherence can occur in photosynthetic processes using femtosecond spectroscopy techniques [2], [20], [33]. Furthermore, it contradicts the idea that quantum coherence can only occur at low temperatures. Likewise, some studies show quantification of the degree of non-Markovianity of quantum coherence in open systems [21], [22], [23], [40], [41]. However, the presence of incoherent maps and the reflux of information from the environment to the system do not allow us to trace all the memory effects inherent in the reservoir system [21], [26], [33]. Formally, the measure of non-Markovianity is

(1) $$N_{C}=-\int_{\frac{d}{dt}|G(t)|>0}\frac{d}{dt}|G(t)|dt,$$

where, G(t) is the solution of the integral equation $\frac{d}{dt}G(t)=-\int_{0}^{t}dt^{\prime }f(t-t^{\prime })G(t^{\prime })$. The kernel $f(t-t^{\prime })$ is given by the correlation function $f(t-t^{\prime })=\mathrm{Tr}\{A(t)A{\left(t^{\prime }\right)}^{†}\rho_{E}\}\exp[iw_{0}(t-t^{\prime })]$ where, $\rho_{E}=(|0\rangle {\langle 0|)}_{E}$ corresponds to the empty state of the reservoir and, *A* is a positive operator. This kernel is generally expressed in terms of the spectral density of the reservoir J(w) as follows

(2) $$f(t-t^{\prime })=\int dwJ(\omega )\exp[i(\omega_{0}-\omega )(t-t^{\prime })].$$

In this way, the exact Eq. (2) can be solved by means of a Laplace transformation according to the spectral density J(w) that is obtained in each particular case.

However, we take the measure of the non-markovianity of the coherence $N_{\gamma }(T)=\int_{t}^{t^{\prime }}(|\gamma (\tau )|-\gamma (\tau ))d\tau$, where $\gamma (t)=-2Re\left(\frac{\overset{˙}{G}(t)}{G(t)}\right)$, and $RHP=-2Re\left(\frac{\overset{˙}{G}(t)}{1-2G(t)}\right)$. Furthermore, we have considered t=0, and a sufficient large $t^{\prime }=400$ ps [1], [2], [33].

### 2.2 Linear response theory

In this section, we have retraced the analysis of the *LRT* in the two-band model [15]. We will focus attention specifically on the quantification of the memory effects of the respective open quantum system in mention, and we highlight and compare theoretical results regarding experimental accessibility. In general, we associate a separable complex Hilbert space with the respective quantum system, reservoir, and interaction. The total Hamiltonian is represented as follows

(3) $$\overset{ˆ}{H}={\overset{ˆ}{H}}_{S}+{\overset{ˆ}{H}}_{R}+{\overset{ˆ}{H}}_{I}+\varrho {\overset{ˆ}{H}}_{\mathrm{ext}}(t)$$

where, ${\overset{ˆ}{H}}_{S}=\sum_{\overset{\to }{p}}{\overset{ˆ}{H}}_{S}(\overset{\to }{p}),{\overset{ˆ}{H}}_{S}=\phi (\overset{\to }{p})+\sum_{\alpha =x,y,z}d_{\alpha }(\overset{\to }{p}){\overset{\to }{\sigma }}_{\alpha }$ is the Hamiltonian of the quantum systems, $\phi (\overset{\to }{p})=\frac{{\overset{\to }{p}}^{2}}{2m}$ is the kinetic energy with the band electron effective mass, **m** and ${\overset{\to }{\sigma }}_{\alpha }$ are the Pauli matrices, $\overset{\to }{p}=(p_{x},p_{y})$ denotes the Bloch wave vector of the electron and $d_{\alpha }(\overset{\to }{p})$ depends on the texture of the material under consideration [15]. Now we consider that ${\overset{ˆ}{H}}_{R}=\sum_{j}\hbar \omega_{j}{\overset{ˆ}{a}}_{j}^{†}{\overset{ˆ}{a}}_{j}$ is the Hamiltonian of the environment, ${\overset{ˆ}{H}}_{I}=\sum_{j}\hbar g_{j}\overset{ˆ}{\sigma }-{\overset{ˆ}{a}}_{j}^{†}+H.c$ stand for the coupling between the system and the environment, *ϱ* stands for the perturbation parameter and ${\overset{ˆ}{H}}_{\mathrm{ext}}(t)=\frac{1}{\varrho }\sum_{\mu }{\overset{ˆ}{P}}_{\mu }D_{\mu }(t)$ describes the coupling of the quantum system to an classical external field, where $D_{\mu }(t)$ denotes the components of the electric displacement field D(t) with *ϱ* being the dielectric permittivity, in linear medium $D_{\mu }(t)=\varrho E_{\mu }(t)$ and ${\overset{ˆ}{P}}_{\mu }=\sum_{j}q_{j}{\overset{ˆ}{r}}_{\mu }^{j}\cdot q_{j}$ and ${\overset{ˆ}{r}}_{\mu }^{j}$ are the charge and positive vector of the electrons [15].

The density operator $\overset{ˆ}{\rho }(t)$ of the total system satisfies,

(4) $$\overset{˙}{\overset{ˆ}{\rho }}(t)=-\frac{i}{\hbar }[\overset{ˆ}{H}(t),\overset{ˆ}{\rho }(t)]\equiv -\frac{i}{\hbar }\overset{ˆ}{L}(t)\overset{ˆ}{\rho }(t).$$

Subsequently, the total density matrix is divided into two parts, as follows,

(5) $$\overset{ˆ}{\rho }(t)={\overset{ˆ}{\rho }}_{0}(t)+\delta {\overset{ˆ}{\rho }}_{ext}(t),$$

where ${\overset{ˆ}{\rho }}_{0}(t)$ is the total density matrix and initial condition $\overset{ˆ}{\rho }(0)={\overset{ˆ}{\rho }}_{0}(0)$. $\delta {\overset{ˆ}{\rho }}_{ext}(t)$ denotes the change of $\overset{ˆ}{\rho }(t)$ due tho the external field. By analogy, the Liouville operator can be divided into

(6) $$\overset{ˆ}{L}(t)={\overset{ˆ}{L}}_{0}(t)+{\overset{ˆ}{L}}_{ext}(t).$$

Secondly, the functions of the environment at zero temperature f(t) and finite temperature $\overset{¯}{f}(t)$ take

(7) $$f(t)=\sum_{j}{\left\|g_{j}\right\|}^{2}e^{-i\omega_{j}t}\equiv \int_{0}^{\infty }J(\omega )e^{-i\omega_{j}t}d\omega ,$$

(8) $$\overset{¯}{f}(t)=\sum_{j}{\left\|g_{j}\right\|}^{2}N(\omega_{j})e^{-i\omega_{j}t}\equiv \int_{0}^{\infty }J(\omega )N(\omega_{j})e^{-i\omega_{j}t}d\omega ,$$

where J(ω) denotes the spectral density of the environment, $N(\omega )=\frac{1}{e^{\frac{\hbar \omega }{\kappa_{B}T}}-1}$ is the average photon number of the environment, and $\kappa_{B}$ the Boltzmann constant. On the other hand, we will calculate the Dephasing Response Factor (*DRF*) in the next section, according to Ref. [15] as follows

(9) $$DRF(w)=\int_{0}^{\infty }dte^{-i\Delta Et}f(t)e^{iwt},$$

where, $\Delta E=E_{m}-E_{n}$ is the energy gap.

### 2.3 Spectroscopy measure

We have used Wolfram Mathematica software for analytical calculations of non-Markovian profiles and numerical integration techniques, the successive approximation method, the Newton-Raphson method, and secant in nonlinear dynamics. Now, we quantify how different a given measured spectrum is from a simulated Lorentzian spectrum; we define a measure of non-Markovian character in terms of a cross-correlation between the measured spectrum and a simulated Markovian spectrum (see Ref. [3], [23] for more details). In doing so, we define

(10) $$D(\omega_{0})=\frac{\int dw{⋀}_{m}(\omega_{0}+\omega ){⋀}_{M}(\omega )}{\int dw{⋀}_{nM}(\omega_{0}+\omega ){⋀}_{M}(\omega )},$$

where ⋀ denotes either the absorption or fluorescence spectrum. ${⋀}_{m}$ denotes the measured spectrum, ${⋀}_{M}$ the simulated Markovian one (κ≥1), ${⋀}_{nM}$ stands for the simulated non− Markovian spectrum (κ≪1). The measure of non-Markovianity is introduced as the convolution of the detected spectrum with the Lorentz shape.

## 3 Results and discussion

In this section we found the degree of non-markovianity for the two-band open quantum system (topological insulators) and calculated the Dephasing Response Factor as a function of frequency [2], [3], [33]. In fact, we easily observe that $N_{C}>0$, $N_{\gamma }>0$, $N_{RHP}>0$, that is, we guarantee the memory effects of the respective dynamics of the open quantum system [21], [23], [27]. First, we focus attention on the spectral density

(11) $$J(\omega )=\frac{1}{\pi }\frac{\Gamma }{{\left(\omega -\omega_{eg}\right)}^{2}+\Gamma },$$

here, $\Gamma =\frac{\lambda k_{B}T}{\hbar \Lambda }$, where *λ* denotes the spectral bandwidth, and $k_{B}$ the Boltzmann constant. However, we substitute Eq. (11) into Eq. (7), we can obtain the correlation function

(12) $$f(t)=\frac{1}{\pi }\int_{0}^{\infty }\frac{\Gamma }{{\left(\omega -\omega_{eg}\right)}^{2}+\Gamma }(\cos(\omega t)-i\sin(\omega t))d\omega$$

Subsequently, we solve the integro-differential equation

(13) $$\frac{d}{dt}G(t)=-\int_{0}^{t}dt^{\prime }f(t-t^{\prime })G(t^{\prime }).$$

Therefore,

(14) $$G(t)=e^{\frac{-t\sqrt{\Gamma }}{2}}\left[\cosh\left(\frac{1}{2}t\Omega \right)+\frac{\sqrt{\Gamma }\sinh\left(\frac{1}{2}t\Omega \right)}{\Omega }\right],$$

(15) $$\gamma (t)=\frac{-2e^{\frac{t\sqrt{\Gamma }}{2}}\left(-\frac{1}{2}\sqrt{\Gamma }e^{-\frac{t\sqrt{t}}{2}}\cosh(\frac{1}{2}t\Omega )+\frac{\sqrt{\Gamma }\sinh\left(\frac{1}{2}t\Omega \right)}{\Omega }\right)}{cosh\left(\frac{1}{2}t\Omega \right)+\frac{\sqrt{\Gamma }\sinh\left(\frac{1}{2}t\Omega \right)}{\Omega }}-\frac{e^{\frac{-t\sqrt{\Gamma }}{2}}\left(\frac{1}{2}\Gamma \cosh\left(\frac{1}{2}t\Omega \right)+\frac{1}{2}\Omega \sinh\left(\frac{1}{2}t\Omega \right)\right)}{cosh\left(\frac{1}{2}t\Omega \right)+\frac{\sqrt{\Gamma }\sinh\left(\frac{1}{2}t\Omega \right)}{\Omega }}$$

where $\Omega =\sqrt{-4\sqrt{\Gamma }+\Gamma }$. We substitute Eq. (14) into Eq. (1). In this sense, we analyzed the quantum coherence measure of non-Markovianity, normalized, by varying the respective spectral width.

**Figure 1:**
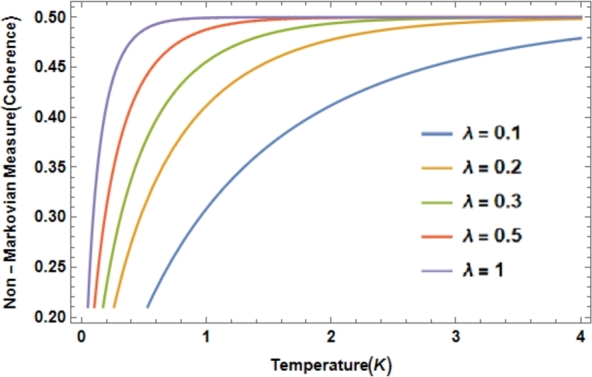
Delineating incoherent non-Markovian dynamics using quantum coherence $N_{C}$, normalized. In addition, we consider *ħ* = 1, *λ* = [0 − 1], Λ = 10.

Fig. 1 shows the respective non-Markovian incoherent dynamical delineation using quantum coherence [3], [31], [33]. In this case, we compare the respective memory effects concerning temperature and varying spectral width for the topological two-band system. We have observed that regardless of the spectral bandwidth of the environment, a convergence in the non-Markovian quantification follows in the respective renormalization. However, this inconsistent measure does not show all the effects of memory; therefore, it should be extended to the non-Markovian quantification of quantum coherences or, failing that, apply nonlinear spectroscopy [3], [33]. We calculate the *DRF*

(16) $$DRF(\omega )=-i(\frac{\frac{\pi }{a(\omega )}-\frac{\pi }{b(\omega )}+i\frac{Log[-i-\Gamma ]}{c(\omega )}+i\frac{Log[\frac{\Delta E-\omega }{-1+i\Gamma }]}{c(\omega )}-i\frac{Log[\frac{i}{-i+\Gamma }]}{d(\omega )}}{2\pi })-i(\frac{-i\frac{Log[\frac{i(\Delta E-\omega )}{-i+\Gamma }]}{d(\omega )}-i\frac{Log[-i+\Gamma ]}{d(\omega )}+i\frac{Log[\frac{-i}{i+\Gamma }]}{c(w)}}{2\pi }),$$

where, a(w)=2+2ΔE−2ω−2iΓ, b(ω)=2+2ΔE−2ω+2iΓ, c(ω)=1+Δ−ω−iΓ, d(ω)=1+ΔE−ω−iΓ, and Δ*E* is the energy gap.

**Figure 2:**
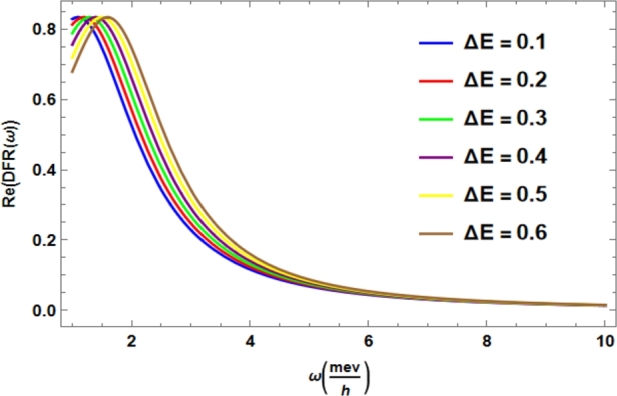
Dephasing Response Factor, normalized. In addition, we consider *ħ* = 1, *λ* = 0.1 *mev*, Δ*E* = [0.1 − 0.6], $\Gamma =0.2\frac{mev}{\hbar }$.

In Fig. 2, we represent the Dephasing Response Factor depends on the strength of coupling between the system and the environment and the difference of two energy bands; in fact, when modulating the bandwidth of the ambient spectrum *λ*, several studies indicate that susceptibility changes from the non-Markovian regime to the Markovian regime, regardless of the form that the Hamiltonian system takes [15].

**Figure 3:**
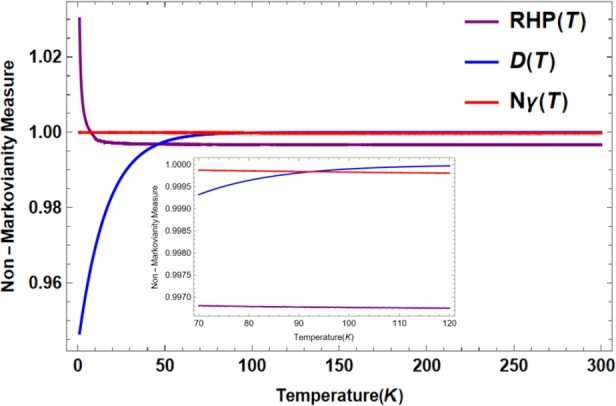
Quantification of the non-Markovian profile for the two-band system (topological insulator), normalized. In addition, we consider *ħ* = 1, *λ* = 0.1, Λ = 10, 0 < *T* ≤ 300*K* where, *T* is the temperature of the reservoir.

In Fig. 3, we compare the three measures $N_{\gamma }(T)$, $N_{RHP}(T)$, and D(T) of non-markovianity for the two-band model described in Ref. [15]. We apply numerical approximation techniques, without introducing auxiliary systems or full knowledge of dynamic maps, to quantify the non-Markovian degree of the $N_{\gamma }(T)$ coherence based on the information flow returned from the two band model.

The non-Markovian quantum coherence curve (red) shows memory effects for low and high temperatures. Thus, the importance of the respective measurement in the loss or conservation of structural properties in two-band open quantum systems in topological insulators is demonstrated.

On the other hand, we incorporate the $N_{RHP}(T)$ measure to optimize memory effects and construct an experimentally accessible D(T) measure supported by nonlinear spectroscopy [3], [20], [21], [23]. In this case, the representation D(T) defines a measure of non-Markovian character in terms of a cross-correlation between the measured spectrum and a simulated Markovian spectrum [3], [21], [23], [31]. Here we show that the measurements $N_{RHP}(T)$, $N_{\gamma }(T)$ and D(T) exhibit memory effects for the two-band model (topological insulators), valid for both low and high temperatures.

In this sense, the optical coherence in topological insulators is affected by the interaction with the respective bath, mediated by the two-band model [15]. Now, according to Ref. [15] the two bands can describe other physical phenomena, for example: descriptions of spin-orbit coupling when the system is a spin-1/2 electron group in solids, and they can describe orbital hybridization when the two bands represent orbitals.

Therefore, $N_{RHP}(T)$ describes the highest degree of non-Markovianity at low temperatures, However, the measurements $N_{\gamma }(T)$, and D(T) gradually show larger memory effects. That is, the strong coupling of the reservoir to the quantum system at sufficiently long times and high temperatures validates the hypotheses of non-Markovianity [1], [2], [33]. Furthermore, the measure D(T) describes memory effects in the superconductivity region and nonlinear spectroscopy problems (see Ref. [3], [15], [23] for more details).

Stepping back, Fig. 1 provides the delineation of the non-Markovian dynamics in the two-band topological model for the measure $N_{C}$ varying spectral width and being experimentally accessible at low temperatures. However, the incoherent maps restrict the respective dynamics for upper limits of frequencies and temperatures [15], [20], [21]. Therefore, such maps should be extended to coherent dynamics, incorporating the measurement of memory effects in quantum coherence (see Ref. [2], [20], [33] for details). On the other hand, Fig. 3 supports non-Markovian quantification as a function of temperature for 0<T≤300K [16], [21], [23]. Furthermore, *DRF* exhibits modulation of the input parameters of the respective spectral density, being compatible at inflection points with respect to the chosen temperature and the energy band of the system [1], [2], [33]. Fig. 2 and Fig. 3 correlate the quantified of memory effects, supported by the Born approximation of Linear Response Theory and nonlinear spectroscopy for the topological two-band open quantum system [23], [31], [33].

## 4 Concluding remarks

The two-band open quantum system for topological insulators is affected by non-Markovian features, mediated by incoherent operations allowed by the quantum coherence measure. On the other hand, the $N_{RHP}(T)$, $N_{\gamma }(T)$ and D(T) measures correctly describe the memory effects in the respective two-band model for topological insulators for 0<T≤300K. In this case, the input parameters of the respective reservoir were calibrated, mediated by nonlinear spectroscopy to avoid information reflows. Furthermore, the *DRF* has been ratified as a calibrating tool to observe the limits of non-Markovian dynamics by varying the spectral density parameters of the two-band system. Finally, the obtained results are experimentally accessible, applicable to quantum statistics, condensed matter, resource theory and quantum chaos.

## Additional information

No additional information is available for this paper.

## Funding

This research did not receive any specific grant from funding agencies in the public, commercial, or not-for-profit sectors

## CRediT authorship contribution statement

**H. Triviño:** Writing – original draft, Software, Methodology, Investigation, Data curation, Conceptualization. **F. Mesa:** Writing – review & editing, Writing – original draft, Visualization, Validation, Supervision, Project administration, Investigation, Formal analysis. **VA. Ballesteros:** Writing – original draft, Visualization, Supervision, Resources, Project administration, Investigation, Funding acquisition.

## Declaration of Competing Interest

The authors declare the following financial interests/personal relationships which may be considered as potential competing interests: Fredy Giovanni Mesa reports was provided by Fundación Universitaria Los Libertadores. Fredy Mesa reports a relationship with Fundación Universitaria Los Libertadores that includes: employment. If there are other authors, they declare that they have no known competing financial interests or personal relationships that could have appeared to influence the work reported in this paper.

## Data Availability

The data will be made available upon request.
